# Supplementary material for: The genome sequence of the Marsh Pennywort, Hydrocotyle vulgaris L. (Apiales: Araliaceae)
Source: Wellcome Open Res. 2025 Jul 28;10:370. [Version 1] doi: 10.12688/wellcomeopenres.24582.1 (PMC12421231; doi:10.12688/wellcomeopenres.24582.1)
Supplement: Peer review history

# Reviewer report: Reviewer response for version 1

**DOI:** 10.21956/wellcomeopenres.27091.r130599

**Published:** 2025-09-09

**Authors:** Bimal K Chetri

**Affiliations:** 1 Indian Institute of Technology, Guwahati, Assam, India

The assembly is of high quality and clearly valuable, but the manuscript would benefit from an explicit clarification of how the haploid genome size estimate (~467 Mb) and the flow cytometry estimate (~1,050 Mb) relate to the final haplotype assemblies (~870 Mb and ~863 Mb). Since *Hydrocotyle vulgaris* is an octoploid (2n = 96), a short statement connecting polyploidy to these size differences would make the Data Note more transparent and easier for downstream users to interpret.

Are sufficient details of methods and materials provided to allow replication by others?

Yes

Is the rationale for creating the dataset(s) clearly described?

Yes

Are the datasets clearly presented in a useable and accessible format?

Yes

Are the protocols appropriate and is the work technically sound?

Yes

Reviewer Expertise:

Plant Genomics

I confirm that I have read this submission and believe that I have an appropriate level of expertise to confirm that it is of an acceptable scientific standard.

# Reviewer report: Reviewer response for version 1

**DOI:** 10.21956/wellcomeopenres.27091.r130600

**Published:** 2025-09-09

**Authors:** Peng-Fei Ma

**Affiliations:** 1 Chinese Academy of Sciences, Kunming, China

This study reported a high-quality genome assembly of a small aquatic perennial, Hydrocotyle vulgaris. This species is native to Europe and Northern Africa, and particularly widespread in Britain and Ireland. It even becomes an invasive plant in China. The genome sequences reported here may be helpful for understanding the clonal reproduction of this species  and potentially control of it. I have two questions. In the results, a heterozygosity rate of 37.71%  was reported and please check this number as it is so high. The other one is that the assembled quality of Haplotype 2 seems better than Haplotype 1, and why  Haplotype 1 was used for Hi-C scaffolding?

Are sufficient details of methods and materials provided to allow replication by others?

Yes

Is the rationale for creating the dataset(s) clearly described?

Yes

Are the datasets clearly presented in a useable and accessible format?

Yes

Are the protocols appropriate and is the work technically sound?

Yes

Reviewer Expertise:

Plant genomics

I confirm that I have read this submission and believe that I have an appropriate level of expertise to confirm that it is of an acceptable scientific standard.

# Reviewer report: Reviewer response for version 1

**DOI:** 10.21956/wellcomeopenres.27091.r128829

**Published:** 2025-08-30

**Authors:** Sunil Kumar Sahu

**Affiliations:** 1 BGI Research, Shenzhen, China

This manuscript presents a high-quality, chromosome-scale genome assembly for *Potentilla sterilis* (Barren Strawberry), generated as part of  Darwin Tree of Life (DToL) project. The assembly is robust, utilizing modern long-read and Hi-C scaffolding technologies, and meets high-quality standards. The resource will be a valuable addition to the genomic resources available for the Rosaceae family and for studying polyploidy in plants. The manuscript is generally well-written and follows a standard format for data notes.

Are sufficient details of methods and materials provided to allow replication by others?

Yes

Is the rationale for creating the dataset(s) clearly described?

Yes

Are the datasets clearly presented in a useable and accessible format?

Yes

Are the protocols appropriate and is the work technically sound?

Yes

Reviewer Expertise:

Plant genomics and evolution

I confirm that I have read this submission and believe that I have an appropriate level of expertise to confirm that it is of an acceptable scientific standard.
